# Decreased subsets of T lymphocytes CD8 in patients with IgE food allergy

**DOI:** 10.1186/1939-4551-8-S1-A272

**Published:** 2015-04-08

**Authors:** Isaac Azevedo Tenorio, Aderbal Sabra, Selma Sabra

**Affiliations:** 1 Unigranrio, Brazil

## Objective

To study the decrease of CD8 T lymphocytes in patients with FoodAllergy (FA) of the IgE type associating with its clinical manifestations.

## Method

Analysis of 16 records of patients selected as having the IgE Food Allergy , who presented the CD4 / CD8 ratio, greater than 3, by reduction of CD8 bellow the average and evaluating its clinical presentation of FA.

## Material and methods

Of these 16 records, 11 were girls (68.75%) and 5 were boys (31.25%).

## Results

8 children had gastrointestinal imediated hypersensitivity (42.10%), 6 had increased this ratio due to hives (31.57%), 3 had rhinitis (15.78%), 1 asthma (5.26 %) and 1 had anigioedema (5.26%). The main complaints of these children were also analyzed. The most frequent complaints were skin with 9 reports (33.33%), followed by constipation with 7 (25.94%) and respiratory complaints with 6 (22.22%). There were also 5 reports of accute diarrhea (18.51%), 3 with abdominal pain and vomiting, 3 with weight loss and 1 with reflux. In this study of the 16 children with CD4 / CD8 ratio high, 15 were born by cesarean delivery.

## Conclusion

The analysis of the records of patients with IgE FA and high CD4 / CD8 ratio has shown that these patients will reflect in the clinic with symptoms of skin, respiratory and gastrointestinal disorders. Of these, we note that the most common symptoms are skin complaints (33.33%) and the most common disease that leads to decreased CD8 is the Immediate Gastrointestinal Hypersensitivity (42.10%). Hardest hit were the GALT systems and SALT. 15 of the 16 studied cases were born by cesarean delivery.

